# r3Cseq: an R/Bioconductor package for the discovery of long-range genomic interactions from chromosome conformation capture and next-generation sequencing data

**DOI:** 10.1093/nar/gkt373

**Published:** 2013-05-11

**Authors:** Supat Thongjuea, Ralph Stadhouders, Frank G. Grosveld, Eric Soler, Boris Lenhard

**Affiliations:** ^1^Computational Biology Unit, Uni Computing, Uni Research AS, N-5020 Bergen, Norway, ^2^Department of Molecular Biology, University of Bergen, N-5020 Bergen, Norway, ^3^Department of Cell Biology, Erasmus Medical Center, 3015GE Rotterdam, The Netherlands, ^4^Center for Biomedical Genetics and Cancer Genomics Center, Erasmus Medical Center, 3015GE Rotterdam, The Netherlands, ^5^Laboratory of Hematopoiesis and Leukemic Stem Cells (LSHL), CEA/INSERM U967, Fontenay-aux-Roses, France, ^6^Department of Molecular Sciences, Institute of Clinical Sciences, Faculty of Medicine, Imperial College London, Hammersmith Hospital Campus, Du Cane Road, London W12 0NN, UK, ^7^MRC Clinical Sciences Centre, Hammersmith Hospital Campus, Du Cane Road, London, W12 0NN, UK and ^8^Department of Informatics, University of Bergen, N-5008 Bergen, Norway

## Abstract

The coupling of chromosome conformation capture (3C) with next-generation sequencing technologies enables the high-throughput detection of long-range genomic interactions, via the generation of ligation products between DNA sequences, which are closely juxtaposed *in vivo*. These interactions involve promoter regions, enhancers and other regulatory and structural elements of chromosomes and can reveal key details of the regulation of gene expression. 3C-seq is a variant of the method for the detection of interactions between one chosen genomic element (viewpoint) and the rest of the genome. We present *r3Cseq*, an R/Bioconductor package designed to perform 3C-seq data analysis in a number of different experimental designs. The package reads a common aligned read input format, provides data normalization, allows the visualization of candidate interaction regions and detects statistically significant chromatin interactions, thus greatly facilitating hypothesis generation and the interpretation of experimental results. We further demonstrate its use on a series of real-world applications.

## INTRODUCTION

The availability of complete sequenced genomes and increasingly deep coverage of transcriptomes has led to the successful annotation of protein-coding genes and a growing number of non-coding RNA genes in eukaryotic genomes. The mechanisms involved in regulating these genes in different cell types, in various developmental and differentiation processes, and under different environmental conditions are under intensive investigation, recently accelerated by high-throughput methods for the detection of promoters and regulatory elements (1–3). One of the key tasks in integrating data on gene expression with the location and activity of regulatory elements is to elucidate which regulatory elements interact with which gene promoters, and with which other regulatory elements, in a particular cellular context. Much of the early progress in studying the regulatory elements that act directly on distant target genes via physical interactions was made using DNA fluorescence *in situ* hybridization (4). However, DNA fluorescence *in situ* hybridization can only be used for a limited number of DNA loci at a time, and it provides only low-resolution data. The advent of the chromosome conformation capture (3C) technique (5), which generates novel ligation products between DNA sequences that are closely juxtaposed in the nuclear space *in vivo*, has led to many long-range genomic interactions detected at high resolution. A key study during the development of 3C showed that the looped conformation between the β-globin genes and the locus control region (LCR) was specific to erythroid cells where the genes are expressed, suggesting that promoter–enhancer contacts may be required for transcriptional regulation (6). The 3C method has been widely used to detect chromosomal interactions in mammalian cells. However, this technique is still low-throughput, as it relies on locus-specific polymerase chain reaction (PCR) primers and can only be used to interrogate chromatin interactions between pairs of pre-selected sequences. Therefore, many efforts have been made to develop protocols for high-throughput 3C-based analyses that allow the identification of many interactions in parallel [for review see (7)]. The resulting methods include for instance (i) 3C-on-chip (4C) (8) and 4C-seq (9), which can be used to identify the genome-wide interactions with a specific fragment of choice (a ‘viewpoint’), (ii) 3C-carbon-copy (5C) (10,11), which probes interactions with many viewpoints within a confined genomic region (typically ∼1 Mb), or (iii) Hi-C (12), the latter being able to identify interactions between all genomic sites. Each of these methods has advantages and disadvantages, and the specific choice of method depends on the type of question to be answered.

We have previously developed a 3C-seq protocol (13,14) based on an adaptation of the 4C-method (8) to next-generation Illumina sequencing. This protocol generates a vast amount of data consisting of millions of reads from regions of genomic interaction and requires a set of bioinformatics methods and tools to facilitate data preprocessing and data analysis, interpretation and visualization of candidate interaction regions. Currently, there are few tools available for 3C-seq data analysis (9,15). These tools only provide window-based analysis methods, which have the disadvantage of using an arbitrary window size that might limit the identification of interaction regions to within a certain size range. In addition, these tools do not facilitate the analysis of replicate experiments. To address these needs, we have developed an R/Bioconductor package called *r3Cseq*, a publicly available bioinformatics software package for 3C-seq studies, to perform the analysis of data generated by 3C-seq technology. The package provides a comprehensive workflow that starts with the aligned reads and ends with an interpretable visualization of regions of interaction. It can analyze data from various experimental designs, with or without a control experiment, and it supports in-depth data analysis of replicate experiments. It enables 3C-seq data normalization and statistical analysis for the identification of *cis* and *trans* interactions (i.e. interactions between regions on the same chromosome and interactions between different chromosomes, respectively), using both restriction fragment-based and window-based methods. These functions will allow scientists to compare different ways of analyzing their data set and select the most suitable analysis for the interpretation of their data. Finally, *r3Cseq* produces a range of plots specifically designed for the visualization of genomic regions that physically interact with the selected genomic regions of interest. The output generated by *r3Cseq* consists of simple text and *bedGraph* (16) files compatible with visualization using other tools, such as the UCSC Genome Browser (16) and IGV (17).

## MATERIALS AND METHODS

### Principles of the 3C-seq procedure and *r3Cseq* data analysis workflow

**Figure 1.**
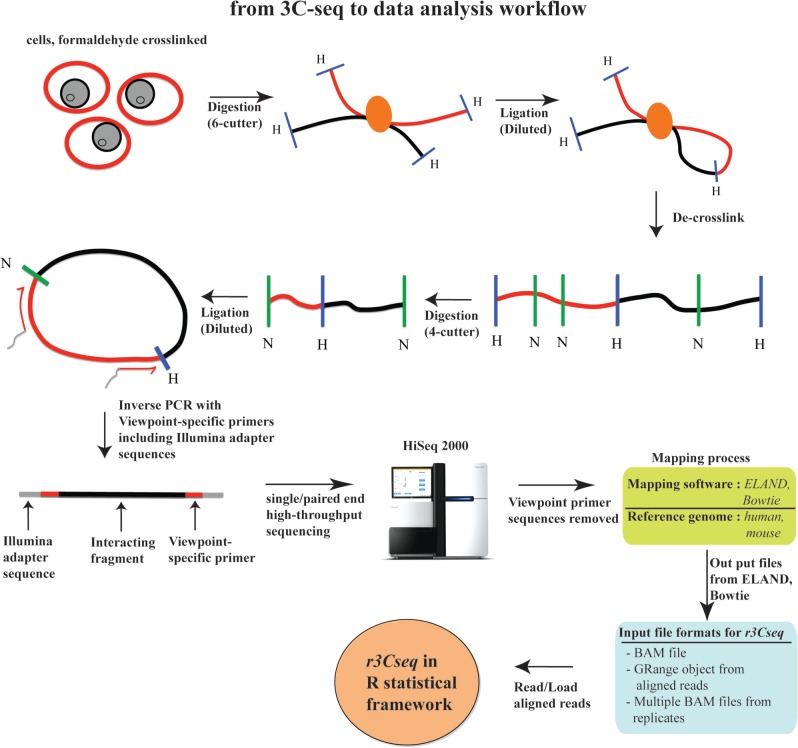
3C-seq experimental procedures and data analysis workflow. Formaldehyde cross-linked chromatin is digested with a six-cutter restriction enzyme and ligated under dilute conditions. After de-cross-linking, DNA is digested with a four-cutter enzyme and again ligated under dilute conditions to create small circular fragments representing individual ligation events. Inverse PCR using viewpoint-specific primers containing Illumina sequencing adapters is used to generate a viewpoint-specific 3C-seq library. After high-throughput sequencing, reads are trimmed and mapped to the reference genome, after which they are loaded into the *r3Cseq* software.

**Figure 2.**
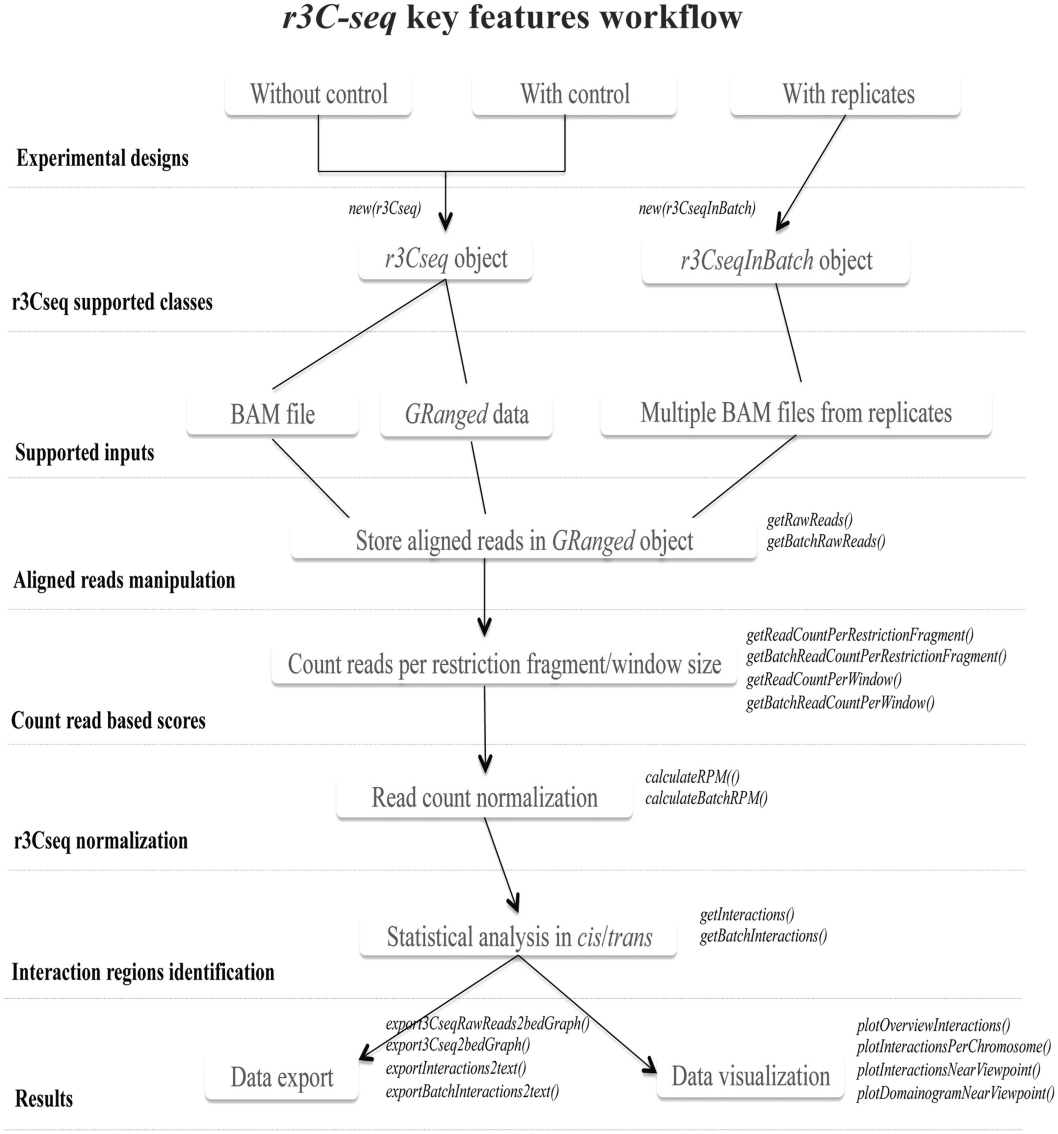
A summary of the *r3Cseq* analysis pipeline. The main features and the sequential order of operations are shown in the flow chart. In-depth discussion of the different operations and functions can be found in the ‘Materials and Methods’ and ‘Results’ sections.

The 3C-seq experimental procedure is outlined in Figure 1, and the subsequent *r3Cseq* data analysis workflow is shown in Figure 2. Isolated cells are treated with a cross-linking agent to preserve *in vivo* nuclear proximity between DNA sequences. The DNA isolated from these cells is then digested using a primary restriction enzyme, typically a 6-bp cutting enzyme, such as HindIII, EcoRI or BamHI. The digested products are then ligated under diluted conditions to favor intra-molecular over inter-molecular ligation events. This digested and ligated chromatin yields composite sequences representing (distal) genomic regions that are in close physical proximity in the nuclear space. The digested and ligated chromatin is then de-cross-linked and subjected to a second restriction digest using a four-cutter (e.g. NlaIII or DpnII) as a secondary restriction enzyme to decrease the fragment sizes. The resulting digested DNA is then ligated again under diluted conditions, creating small circular fragments. These fragments are inverse PCR amplified using primers specific for a genomic region of interest (e.g. promoter, enhancer or any other element potentially involved in long-range interactions), termed the ‘viewpoint’. The amplified fragments are then sequenced using massively parallel high-throughput sequencing. The 3C-seq procedure produces DNA molecules consisting of viewpoint-specific primers followed by sequences derived from the ligated interacting fragments. These need to be trimmed *in silico* to remove the primer and viewpoint sequence, thus leaving only the captured sequence fragments for mapping (14). After trimming, reads are mapped against a reference genome using alignment software, such as Bowtie (18).

Our *r3Cseq* package has been developed in the R statistical framework (19) as part of Bioconductor (20). It uses binary alignment/map (BAM)-aligned read files as input (21), which are generated by commonly used alignment software and carries out operations, such as class initialization, counting aligned reads per restriction fragment or per window size, read count normalization, statistical analysis of interactions in both *cis* and *trans*, data visualization and data export of the identified contacting regions. Figure 2 shows the main features and the sequential steps of the *r3Cseq* pipeline.

### Data normalization

Current normalization methods for next-generation sequencing data have shown that the read count distribution per region observed in RNA-seq, chromatin immunoprecipitation coupled with high-throughput sequencing (ChIP-seq) and Cap Analysis of Gene Expression (CAGE) experiments approximates a power-law distribution (22,23). To investigate whether this is also the case for 3C-seq data, we analyzed 11 published samples of 3C-seq data generated using different mouse cell types, restriction enzymes and viewpoints (22,23). We observed that, for all samples, read count distributions per restriction fragment and per 5-kb window size approximately fit a power-law distribution (Supplementary Figures S1A and S2A). The slopes of the power-law curves were similar across samples, whereas the read counts varied depending on the sequencing depth. We, therefore, adapted a method originally developed for normalizing deepCAGE data (23). For each sample, we fitted the reverse-cumulative distribution of reads per region to a power-law distribution. To do this, we first filtered out data to remove regions with <50 read counts. We also excluded the viewpoint from the analysis. A frequency table with the distribution of read counts per region was then generated for each sample. This frequency table served as the input for a simple linear regression model to obtain the slope and offset values for all samples. As expected, the offset values significantly vary depending on sequencing depth, whereas the fitted slope values vary within a small range. The fitted slope value across all samples was −1.35 (±0.2) on average. To normalize the read count per restriction fragment or per window size, we developed R functions to implement the formula described in the deepCAGE method by choosing a power-law reference distribution with an exponent of α = −1.35 and an *n_0_* = 1 million offset. The normalization functions are used to transform the read counts from all samples into normalized reads per million (RPM). Supplementary Figures S1C and S2C show the read count distribution after normalization. We also implemented a function to calculate a simpler defined RPM measure for genomic regions, using the number of aligned reads observed at the particular restriction fragment or window divided by the total number of the aligned reads, multiplied by 1 million. Supplementary Figures S1B and S2B show the read count distributions after normalization using this simple RPM calculation. As expected, the reverse-cumulative fitted values from the power-law distributions revealed a better fit of the normalized values for all samples as compared with the simple RPM calculation. In addition, plots depicting the log2 intensity ratio (M) versus the average log2 intensity values (A) between two samples in different experimental conditions exhibited better scaling. Here, the loess red line is close to M = 0 when using a reverse-cumulative fitting normalization to fit the global dependence between the M–A values, when compared with either no normalization or the simple RPM normalization (Supplementary Figure S3A and B). Furthermore, the log2 ratio of interaction regions between two samples located close to the viewpoint (within ±200 kb, red dots) is not strongly affected after applying the reverse-cumulative fitted values normalization, as the majority of these are in a range of ±3 of the log2 ratio. We, therefore, used the normalized values fitted by the reverse-cumulative of the power-law distribution as the quantitative interaction signals for the fold change calculations to compare interaction intensities between two experimental conditions (see later in the text). The reverse-cumulative fitted values of the power-law normalization method described in this study were implemented as an improvement over the simple RPM normalization that is most often used in count data analysis to remove bias because of unequal sequencing depth. In this study, we demonstrated that this method performs better than those not applying normalization or those applying the simple RPM normalization. However, normalization techniques are still immature for most types of count data generated by next-generation sequencing technologies. Although in-depth development of such methods for 3C-seq is beyond the scope of this study, the *r3Cseq* package can easily be expanded with new normalization methods as they become available.

### Identifying *cis*-interactions from 3C-seq experiments

Published 3C-based studies (8,9,24,25) have shown that interaction intensities are highest around the viewpoint, as DNA sequences near the viewpoint have an increased chance of being non-specifically tethered to the viewpoint during chromatin cross-linking. Interaction signals gradually decrease with increasing distances away from the viewpoint and can only be sporadically captured on other chromosomes. To determine the significant interaction regions of a given viewpoint, we applied a background scaling method to correct for interactions that are simply a consequence of short genomic distance to the viewpoint. We determined the relationship of 3C-seq signals of genomic regions located on the *cis* chromosome by ranking the read count per region based on the relative distance to the viewpoint. The non-parametric regression cubic smoothing spline algorithm implemented in R was then applied with smoothing parameter set between 0.06 and 0.4 (which can be changed by the user). The software uses the smoothing parameter 0.1 by default, as this value exhibits the most suitable steady degree of smoothing (Supplementary Figure S4). We assumed that a relatively small fraction of detected interactions would significantly interact with the given viewpoint. We thus used the average scaled interaction signals as the expected 3C-seq signal for a given genomic distance. 3C-seq signals in *cis* were then transformed into a *Z*-score using the ‘(*obs-exp)*/*SD*’ formula, where *obs* is the observed interaction signal found on the *cis* chromosome, *exp* is the scaled interaction signal for a specific genomic distance and *SD* is the standard deviation of the residual values ‘(*obs-exp)*’. *P*-values can then be assigned to each *Z*-score and transformed into a *q*-value for false discovery rate (FDR) analysis using the *qvalue* package from Bioconductor (20), with a 0.05 FDR level (which can be changed by the user) and using bootstrap as the selected method [*qvalue*(*interactions.p.values, fdr.level = 0.05, pi0.method = ‘bootstrap’*)]. The method we applied to identify interactions in *cis* has successfully been used for 5C data analysis (26), and a similar method has been used for the detection of interactions in 4C data analysis (8,15). Figure 4 shows the analysis results of two data sets using the mouse *Myb* promoter as viewpoint, showing that this method successfully identifies 3C-seq interaction regions in *cis*. These experiments were performed under two experimental conditions: (i) fetal liver (FL) erythrocytes expressing high levels of *Myb* and (ii) fetal brain (FB) cells expressing low levels of *Myb* (24). See the ‘Results’ section for an in-depth discussion of this analysis.

### Identifying *trans*-interactions from 3C-seq experiments

To identify interaction regions in *trans*, we applied a similar formula as described for the identification of interactions in *cis*. It is not necessary to scale the *trans*-signal data, as there is no proximity bias for the interaction signals found on the *trans* chromosomes. We assumed that captured *trans*-interactions would have higher interaction signals than the mean of global interaction signals. We, therefore, transformed the detected interaction signals into a *Z*-score using the ‘(*obs-exp)*/*SD*’ formula, where *obs* is the observed interaction signal found in the whole data set (excluding regions located within ±100 kb around the viewpoint), *exp* is the mean interaction signal for the whole data set and *SD* is standard deviation of the whole data set. Procedures similar to those used for *cis*-interactions were then performed to transform the *trans* interaction signals into statistical interaction scores (*P*- and *q*-values). Applying this method to the *Myb* promoter data sets (24) (see ‘Results’ section) resulted in the identification of several significant interaction regions in *trans* (see Supplementary Figure S5C for an example set of interactions detected in *trans*).

### Analysis of 3C-seq replicate experiments

To investigate 3C-seq data reproducibility among replicates, we performed additional 3C-seq experiments using the *Myb* promoter as the viewpoint in FL erythrocytes and FB cells (3C-seq data are available at http://r3cseq.genereg.net). When considering the entire data set, including signals with low read counts, interacting regions (≥1 RPM, calculated from restriction fragment-based, 5- and 10-kb window-based sizes) across the whole genome in general show low reproducibility, as low intensity signals in 3C-based methods are likely the product of random contacts between DNA fragments (9,15). Remarkably, reproducibility (defined as the percentage of detected interactions present in both replicates against all detected interactions) is extremely low in *trans* (<1% of detected interactions were reproducible, Supplementary Table S1); interactions found in *trans* almost always exhibit very low read counts and are, therefore, likely to be caused by random ligation events. However, in *cis*, reproducibility is significantly higher (17–40%, Supplementary Table S1) and improves when larger window sizes are used for interaction detection. As the most robust interaction regions are invariably located in *cis*, we next checked the reproducibility of high signal interaction regions (≥500 RPM within ±500 kb relative to the viewpoint) and observed that they are highly reproducible (50–90%, Supplementary Table S2), indicating that 3C-seq reliably reveals local chromatin structure around the viewpoint. However, basic analysis of read count data across replicates, such as those implemented in DESeq (27) and edgeR (28), which require overall high reproducibility within the entire data set, is not suitable for data analysis on 3C-seq replicate data sets, especially if one is interested in very long-distance interactions (including inter-chromosomal interactions). To determine significant interactions among replicates, we first performed r3Cseq data analysis for each individual sample. We then combined the detected interactions across biological replicates, providing ‘union’ and ‘intersection’ options for this purpose. The union method combines all significant interactions across samples, whereas the intersection method takes only significant interactions present across all samples into account. Read counts and RPM values across samples are averaged to obtain representative values for the final list of detected interactions. The assigned *P*-values across samples are combined using Fisher’s combined probability test as implemented in R (29), and *q*-values are calculated using the *qvalue* package with FDR level 0.05 (which can be changed by the user), using bootstrap as the selected method.

## RESULTS

### Functionality available in *r3Cseq*

The *r3Cseq* package was built on and extends the functionality of the Bioconductor packages *BSgenome*, *GenomicRanges*, *Rsamtools* and *rtracklayer* (30). It contains functions for the following groups of tasks:

#### Importing aligned reads

*r3Cseq* can read BAM files and converts this file and its related information into an object-oriented core class for the *r3Cseq* package. *r3Cseq* can also load aligned reads from the *GRanges* object generated by the *GenomicRanges* package in R. A detailed description of input parameters can be found in the *r3Cseq* software documentation.

#### Data processing

After class initialization*,* processing functions *getRawReads*, *getBatchRawReads*, *getReadCountPerRestrictionFragment*, *getReadCountPerWindow*, *getBatchReadCountPerRestrictionFragment* and *getBatchReadCountPerWindow* are performed*.* The getRawReads function retrieves aligned reads from BAM files and transforms them to GRanges objects that can be stored in an r3Cseq object, whereas getRawReadsInBatch processes the data in batch mode and stores the aligned reads GRanges in R files (.rdata). To count the number of reads used for further analysis, *r3Cseq* provides two ways to count the number of reads per region; (i) count the number of reads per restriction fragment (using the *getReadCountPerRestrictionFragment* function) and (ii) count the number of reads per non-overlapping defined window (using the *getReadCountPerWindow* function), whereas the *getBatchReadCountPerRestrictionFragment* and *getBatchReadCountPerWindow* functions perform the same analysis for replicate data sets. These functions provide options for counting all reads or only the informative reads (i.e. those that are mapped exactly adjacent to restriction sites). The latter method will exclude reads generated from inappropriately digested chromatin by the restriction enzyme and randomly sequenced DNA fragments (see *r3Cseq* software documentation).

#### Data normalization and the identification of interacting regions

*calculateRPM* and *calculateBatchRPM* are functions that normalize the number of RPM for each restriction fragment or window. Users can select different RPM calculation methods, as described in the ‘Materials and Methods’ section, by defining the normalization method parameters (see *r3Cseq* software documentation). After normalization, the *getInteractions* and *getBatchInteractions* functions are used to calculate *Z*-scores, estimate *P*-values and assign *q*-values to detect significant interactions, respectively. For the *getBatchInteractions* function, users can define the selected combine-method parameter for the detection of interactions across replicates (‘*union*’ and ‘*intersection*’).

#### Visualization

The *plotOverviewInteractions*, *plotInteractionsNearViewpoint*, *plotInteractionsPerChromosome* and *plotDomainogramNearViewpoint* functions are provided for visualization of the interaction regions, taking advantage of the powerful plotting facilities in R. Supplementary Figure S4 shows examples of the plots generated by these functions, allowing users to explore the interaction regions of their data sets.

#### Data export

The *exportInteractions2text*, *exportBatchInteractions2text* and *export3Cseq2bedGraph* functions are used to export the analysis results to tab-delimited text files. The identified interaction regions can be exported into the *bedGraph* format, which can be easily uploaded to the UCSC Genome Browser (16) and IGV (17) for further visualization and exploration.

#### Annotation of interactions

The *getExpInteractionInRefseq* and *getContrInteractionInRefseq* functions provide a list of candidate genes, which contain significant interaction signals in their proximity. Here, proximity is defined by input parameters that specify the relative distances to the start and end positions of genes (see *r3Cseq* software documentation).

#### Preparing a final report

The *generate3CseqReport* function can be used to export all results of the analysis, including plots and text files, into a PDF file that can be used for data interpretation and publication.

## A PROOF OF PRINCIPLE ANALYSIS USING *r3Cseq*

As a proof of principle analysis, we used the *r3Cseq* package to characterize long-range interactions at the mouse β-globin locus using our 3C-seq and ChIP-seq data from Soler *et al.* (13).

The chromosomal architecture of the β-globin locus has been studied intensively and serves as an excellent test case for *r3Cseq* functionality. Previous studies have shown that on activation, the β-major gene (β-maj) from the β-globin locus engages in long-range interactions with upstream regulatory sites (forming the LCR) located 40–60 kb away (31–33). We obtained our 3C-seq data (13) using the β-major globin gene (β-maj) promoter as a viewpoint and applied the *r3Cseq* package for data analysis. 3C-seq experiments were performed in two cell types from the mouse 12.5 dpc embryo: (i) cells expressing the β-globin genes below detection level (FB) and (ii) cells expressing very high levels of β-globin (FL) (9,13). As interactions between the LCR and the β-globin genes have not been observed in FB cells, this experiment served as a negative control to allow the identification of erythroid-specific interactions within the β-globin cluster.

**Figure 3.**
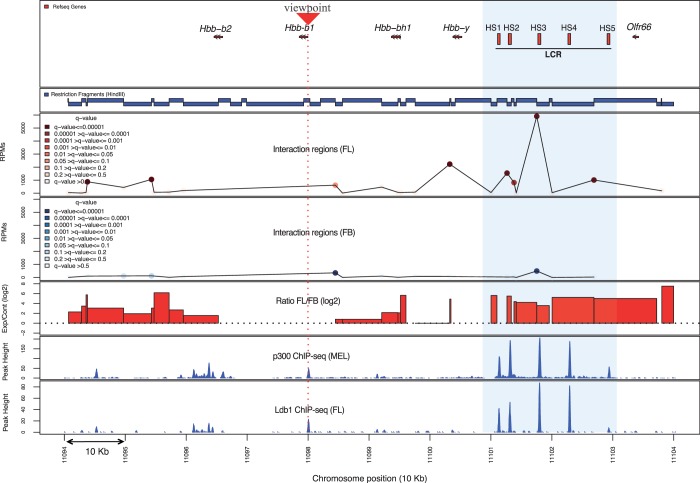
A proof of principle 3C-seq/*r3Cseq* analysis on the well-characterized β-globin locus. Gene locations are shown at the top followed below by a map of restriction fragments. The line plots show overall detected *cis*-interaction signals (40 kb up- and 60 kb downstream of the viewpoint) with the β-major promoter in both FL and FB cells. High signals on the viewpoint fragment or the immediately adjacent fragments were excluded. Color gradients represent the range of significant interaction signals (*q*-value). The bar plot represents the ratio (log2) of normalized signal between FL and FB. The light blue box highlights the LCR region with its hypersensitive sites (HS1-5), coinciding with several FL-specific significant interaction regions and binding sites of transcription factor complexes.

Short reads generated by Illumina sequencing of 3C-seq libraries were mapped to the mouse genome (NCBI37/mm9) using the Bowtie aligner. Mapping files were then analyzed using *r3Cseq* to identify candidate interacting regions and to generate plots for data visualization. As expected, regions identified as interacting in both tissues were found most frequently in *cis* on chromosome 7, where the β-maj viewpoint is located. The interactions predominantly map relatively close to the viewpoint fragment (within 125 kb up- and downstream). However, only in FL erythrocytes, robust interaction regions were detected in the region 40–60 kb upstream of the β-maj gene, corresponding to the location of the LCR. We focused our analysis on this area: the outcome is summarized in Figure 3. In the LCR region, interaction signals detected in FL erythrocytes are much stronger than those observed in FB. These strong interaction regions are statistically significant, with *q* ≤ 0.01. Reassuringly, they coincide with the binding regions of transcriptional co-activator p300 and the Ldb1 transcription factor, known to be involved in enhancer function and globin gene regulation. This confirms the presence of a looping structure, placing the LCR in close proximity to the β-maj gene promoter, and it shows that the sites of long-range interactions coincide with sites of regulatory factor binding (13). *r3Cseq* promptly provides results for both restriction fragment and window-based analysis. We observed that the significant interactions detected from individual restriction fragments and 5- and 10-kb window-based regions in FL are similar. However, the strongest interaction signal is positioned slightly differently depending on the window range; fragment-based analysis detects the strongest signal at the third hypersensitive site (HS3) of the LCR, whereas 5- and 10-kb window-based analysis positions the peak interaction signal at HS2 of the LCR (Supplementary Figure S6). This discrepancy is an obvious consequence of the choice of window size. On the other hand, log2 fold change calculations (FL/FB) show a robust FL-specific interaction signal covering all five HSs of the LCR (Figure 3). Although all methods detect an erythroid-specific β-maj–LCR interaction, the differences between these methods can produce subtle differences in interaction profiles. Additionally, *r3Cseq* provides an option for users to use the *getReadCountPerWindow* function with an ‘overlapping’ window option that may correct for any bias from the arbitrary starting position of windowing. Users will, therefore, have to carefully consider this when selecting their analysis parameters. Because of the generally maximized resolution (∼4 kb) and their unbiased nature, we suggest that the interaction signals detected per individual fragment are the most suitable starting point. Window-based approaches will reduce the detected resolution (depending on the selected size range), but they will improve reproducibility, which can be convenient when long-distance *cis*-interactions or *trans*-interactions are of particular interest.

The *r3Cseq* interaction detection method provides a better interaction score (*q-*values) than was used in our previous analysis (13). This analysis did not include background signal correction, which resulted in assigning overly significant interaction scores to low interaction signals (Supplementary Figure S6). Although our previous method detected ∼4000 contacts, our current methods detected ∼600 significant contacts in FL (*q* ≤ 0.05) using the same cut-off.

To show how the *r3Cseq* package can also be used to analyze data sets generated by other laboratories, we used *r3Cseq* to identify long-range interactions at the mouse β-globin locus using data obtained from a recent 4C-seq study (9). We analyzed those data sets for which the β-maj gene promoter was used as the viewpoint (in FL cells), which was produced using a slightly different protocol (9). In this data set, we were able to demonstrate that our detection method can capture strong interaction regions using individual restriction fragments, 2- and 5-kb window-based analysis in the 100-kb β-globin locus domain (Supplementary Figure S7). Preferred contacts within the locus start from the active β-globin gene toward the most distal HS of the LCR (HS5), and the strongest interaction signals in the LCR are predominantly located between HS1 and HS2. These results coincide and highly correlate with the interaction profile reported in the 4C-seq study (9) (Supplementary Figure S7).

Taken together, we have shown that our *r3Cseq* package can be used successfully to analyze 3C-seq data to reveal long-range chromatin interactions that play critical roles in gene regulation.

## APPLICATION TO GENOMIC REGIONS WITH PREVIOUSLY UNCHARACTERIZED INTERACTIONS

To demonstrate that 3C-seq/*r3Cseq* can be further applied to study chromatin interactions in a structurally unexplored locus, we outline how *r3Cseq* was used to analyze 3C-seq data generated to study the chromatin conformation of the mouse *Myb* locus during erythroid development. The 3C-seq and ChIP-seq data used in this demonstration were taken from previously published data (24).

*Myb*, encoding the c-Myb transcription factor, is a key hematopoietic regulator and plays a pivotal role in maintaining a proper balance between erythroid cell proliferation and differentiation (34–36). Previous reports have shown that erythroid transcription factor complexes occupy distinct sites near *Myb* in the *Myb-Hbs1l* intergenic region (13,37,38). ChIP-seq data obtained for the Ldb1 transcription factor complex, which is a key regulator of erythroid development, revealed a binding cluster in a region spanning 60 kb in the *Myb-Hbs1l* intergenic region. *Myb* expression is highly dynamic during the course of erythroid differentiation (39,40). Considering that the Ldb1 complex is required for proper erythroid maturation and is known to regulate genes in a long distance manner, it was hypothesized that the intergenic Ldb1-complex–binding sites represent distal regulatory elements that control *Myb* expression during erythroid differentiation.

**Figure 4.**
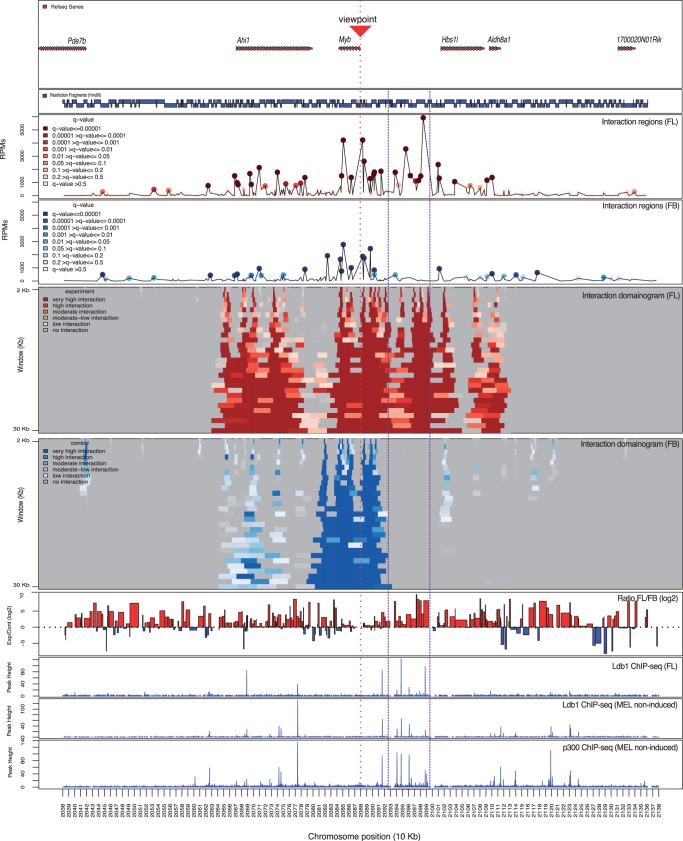
Application of 3C-seq/*r3Cseq* analysis at the *Myb* locus. Gene locations are shown at the top followed below by a map of restriction fragments. The line plots show detected *cis*-interaction regions 500 kb up- and 500 kb downstream of the *Myb* promoter viewpoint in both FL and FB cells. High signals on the viewpoint fragment or the immediately adjacent fragments were excluded. The domainograms show the detected interactions after a window-based analysis (running from 2 to 30 kb) in FL and FB cells. Color gradients of the domainograms represent the interaction signal strength detected for each run of the defined window (transformed *q*-value). The purple dashed box highlights the *Myb-Hbs1l* intergenic region, which shows strong interaction signals coinciding with binding sites of the Ldb1 and p300 transcription factor complexes.

We previously performed 3C-seq experiments using the *Myb* promoter as a viewpoint to investigate whether the Ldb1-complex–binding sites in the intergenic region interact with the *Myb* gene via chromatin looping (24). We used *r3Cseq* to identify and explore the candidate regions, which are interacting with the *Myb* promoter. As for the β-globin locus experiment, 3C-seq was performed on FL erythrocytes (expressing high levels of *Myb*) and FB cells (expressing undetectable levels of *Myb*). The latter was used as a negative control to appropriately link locus structure to gene expression. After mapping, we used *r3Cseq* to analyze the 3C-seq data, and we were able to identify candidate interaction regions, which are shown in Figure 4. In FL erythrocytes, these high interaction signals were found to coincide with the intergenic Ldb1–complex– and p300-binding sites (Figure 4, indicated by the purple dashed box). Interaction frequencies of these regions in FL erythrocytes were statistically significant (*q* ≤ 0.01) and substantially higher than in FB (fold change (log2 ≥ 2), where they were either absent or low. Domainogram plots of interaction regions generated by the *plotDomainogramNearViewpoint* function (using a window-based analysis running from 2 to 30 kb, increasing 1 kb per run) clearly revealed the different intergenic interaction intensity between FL and FB. We also confirmed that these significantly different intergenic interactions between FL and FB can be detected using a different analysis method (9) (Supplementary Figure S8), suggesting that the data generated by our protocol can also be analyzed by other existing tools. Although both analysis methods assign tissue-specific interactions to the intergenic region, *r3Cseq* interactions are more robust when compared with the 4C-seq pipeline (using default parameters, interactions are detected at ∼0.01–0.1 of the median of window coverage). This suggests that *r3Cseq* possesses increased detection sensitivity, at least in this particular case. These results show that analyzing 3C-seq data with *r3Cseq* can identify candidate tissue-specific regulatory regions within a structurally unexplored locus for further experimental investigation. The example data sets and the R codes used to perform the key steps of this analysis are provided at the *r3Cseq* website (http://r3cseq.genereg.net).

We next wanted to test whether our method and software could be used to study the dynamics of long-range chromatin interactions during cellular differentiation. We analyzed 3C-seq data obtained from mouse erythroleukemia (MEL) cells before and after treatment with a differentiation-inducing agent, again using the *Myb* promoter as a viewpoint (24). The 3C-seq data were analyzed using *r3Cseq*. The identified interaction regions are shown in Supplementary Figure S9. In non-induced MEL cells (expressing high levels of *Myb*), interaction regions are similar to those found in FL erythrocytes (Figure 4), often overlapping with Ldb1- and p300-complex–binding sites. Strikingly, on induction of differentiation, these interaction regions showed much lower interaction signals. These results reveal diminished interactions between the promoter and intergenic regulatory regions upon cellular differentiation, coinciding with the downregulation of *Myb* expression and suggesting that these interactions are involved in the regulation of *Myb*. Studying long-range interactions within developmentally regulated loci, exemplified here by the *Myb* locus, is an important application of 3C-seq*/r3Cseq* in exploring the mechanisms of gene regulation.

We next performed 3C-seq replicates for the *Myb* promoter experiment to investigate the reproducibility of the detected interactions across independently prepared samples. As described in the ‘Materials and Methods’ section, we observed that genome-wide detected interaction regions show low reproducibility at the single-fragment level, especially for low signals and inter-chromosomal interactions (Supplementary Table S1). To further investigate such interactions in a set of biological replicate experiments, we used a 20-kb window-based analysis to detect significant interaction across the replicates. Indeed, the *plotOverviewInteractions* function and ‘intersection’ method can remove signals originating from random ligation events present in the single-data set analysis, which mostly occur in *trans* and at long distance sites in *cis* (Supplementary Figure S10A and B). We next determined the genes that are located within the FL significant interaction regions. Using the *getExpInteractionInRefseq* function, we detected 11 genes in the proximity of significant interaction regions (50 kb upstream of the gene start and 5 kb downstream of the gene end), whereas 192 genes were detected in a single-data set analysis in FL erythrocytes (Supplementary Figure S10C and D). As signal reproducibility around the viewpoint is high, genes close to *Myb* where found within this list (*Ahi1*, *Hbs1l* and *Aldh8a1*). Interestingly, of the other genes in relatively close proximity to *Myb* (within 3 Mb), only those that are highly expressed in erythroid cells (*Bclaf1* and *Fam54a*, as determined by RNA-seq in MEL cells, data not shown) are found to interact reproducibly with *Myb*. Five genes located on *trans* chromosomes (*Gm14496*, *Eif4enif1*, *Sfi1*, *Spata5* and *Rit2*) were consistently found in proximity of *Myb*. Whether this observed gene clustering is of any relevance to *Myb* and/or erythroid biology remains unclear, although these observations might prove to be interesting in the context of genetic translocations and transcription factories (41).

## DISCUSSION

We developed the R/Bioconductor package *r3Cseq*, and in this study, we describe its functionality and demonstrate its use and power for the identification of chromatin interaction regions generated by 3C-seq experiments. The software provides functionality for pre-processing, analyzing and visualizing interaction regions with any given viewpoint of interest. The package can process BAM files, which are generated by mapping software, such as Eland and Bowtie. We provided *r3Cseq* with functions to support the BAM file format, as it is a compressed binary file (21), which allows users to perform 3C-seq data analysis on a regular personal computer (CPU ∼2 GHZ with ∼4 GB of random access memory, Supplementary Table S3). However, it is recommended, when possible, to use more powerful computer hardware when performing 3C-seq data analysis on replicate data sets, as this will require more random access memory and storage space.

Our work focused on building the functionality to support data analysis of 3C-seq experiments. We adapted and applied methods used in high-throughput sequencing data analysis for normalization and the detection of significant interaction regions. We applied a fitted reverse-cumulative distribution of reads per region to a power-law distribution as the normalization method, increasing the statistical power of 3C-seq signal detection. This method reveals a better fit of normalized values for 3C-seq data than a simplified RPM calculation. *r3Cseq* still provides functions to support both methods, offering users the choice to use normalized values obtained from each separate method for further analysis. We adapted methods used in previous 4C and 5C studies to detect significant interactions in both *cis* and *trans*. Our method corrects any bias resulting from background interaction signals and assigns an interaction score *(q*-value) directly to a certain restriction fragment or a defined window. Selecting an appropriate window setting for counting reads is critical for 3C-seq data analysis. Both fragment-based and window-based methods have advantages and disadvantages. A fragment-based strategy generally maximizes resolution (∼4 kb) and can identify direct interaction sites in an unbiased way, which might be preferred when specific interaction regions are to be compared with other types of high-resolution data, such as the transcription factor-binding sites identified by ChIP-seq. The outcome of window-based methods depends on the choice of the arbitrary selected window size, subsequently limiting the identification of interaction regions to within that specific size, often reducing the effective resolution. However, the window-based strategy is helpful for detecting large interaction domains, which can significantly promote the identification of novel interaction regions. Larger window sizes show a higher reproducibility (Supplementary Table S1), especially at large distances (>500 kb) from the viewpoint and may also be preferred for replicate data analysis. To facilitate both fragment-based and window-based analysis, *r3Cseq* enables users to easily switch between both types of analysis and promptly provides these results as text files and interpretable plots (see http://r3cseq.genereg.net for more details). *r3Cseq* also supports data analysis of replicate 3C-seq experiments, as it can combine detected interactions across biological replicates to produce a final list of significant interactions. Users can select a union or an intersection operation to obtain the final list of interactions, as described in the ‘Materials and Methods’ section. Both these options are useful, although only the intersection method allows for the detection of truly consistent interaction regions across samples. In summary, we have shown that *r3Cseq* can remove signals originating from random ligation events and provide data normalization, the accurate detection and powerful visualization of both existing and novel significant interaction regions present across multiple biological replicates.

## AVAILABILITY AND IMPLEMENTATION

The *r3Cseq* package has been implemented in R and is available as part of the Bioconductor (www.bioconductor.org) distribution, as version 2.9. As such, it also gives users and software developers the opportunity to extend and customize the pipeline to their needs. We have developed a website to host the *r3Cseq* package, which can be found at http://r3cseq.genereg.net. The website provides downloadable data sets presented in this article and the current version of the *r3Cseq* package (version 1.5.0). The website also describes the R code examples for the 3C-seq data analysis pipeline. Additional guidelines and typical workflows can be found in the package’s vignette in R. The ChIP-seq and 3C-seq data used here were deposited in the sequence read archive (SRA) database. Accession numbers for these data were previously published (9,13,23,24).

## FUTURE DIRECTIONS

In the next version of *r3Cseq* package, more functions will be implemented to allow users to incorporate external data sets, such as ChIP-seq and expression data into the analysis, which will be of great assistance in studying long-range gene regulation.

## SUPPLEMENTARY DATA

Supplementary Data are available at NAR Online: Supplementary Tables 1–3 and Supplementary Figures 1–10.

## FUNDING

EU-FP7 integrated project EuTRACC LSHG-CT-2007-037445 (S.T., R.S., E.S. and F.G.); Dutch Cancer Genomics Center (CGC) and the French Alternative Energies and Atomic Energy Commission (CEA) (to E.S.); Norwegian Research Council (YFF) and Bergen Research Foundation (BFS) (to B.L.). Funding for open access charge: Department of Informatics, University of Bergen (to B.L.).

*Conflict of interest statement*. None declared.

## Supplementary Material

Supplementary Data
