# Supplementary material for: r3Cseq: an R/Bioconductor package for the discovery of long-range genomic interactions from chromosome conformation capture and next-generation sequencing data
Source: Nucleic Acids Res. 2013 May 11;41(13):e132. doi: 10.1093/nar/gkt373 (PMC3711450; doi:10.1093/nar/gkt373)
Supplement: Supplementary Data [file supp_41_13_e132__index.html]

r3Cseq: an R/Bioconductor package for the discovery of long-range genomic interactions from chromosome conformation capture and next-generation sequencing data — r3Cseq: an R/Bioconductor package for the discovery of long-range genomic interactions from chromosome conformation capture and next-generation sequencing data — Supplementary Data 

# r3Cseq: an R/Bioconductor package for the discovery of long-range genomic interactions from chromosome conformation capture and next-generation sequencing data

## Supplementary Data

files

**Files in this Data Supplement:**

- Supplementary Data - pdf file
