# Supplementary material for: r3Cseq: an R/Bioconductor package for the discovery of long-range genomic interactions from chromosome conformation capture and next-generation sequencing data
Source: Nucleic Acids Res. 2013 May 11;41(13):e132. doi: 10.1093/nar/gkt373 (PMC3711450; doi:10.1093/nar/gkt373)
Supplement: Supplementary Data [file supp_gkt373_nar-03249-met-k-2012-File006.pdf]

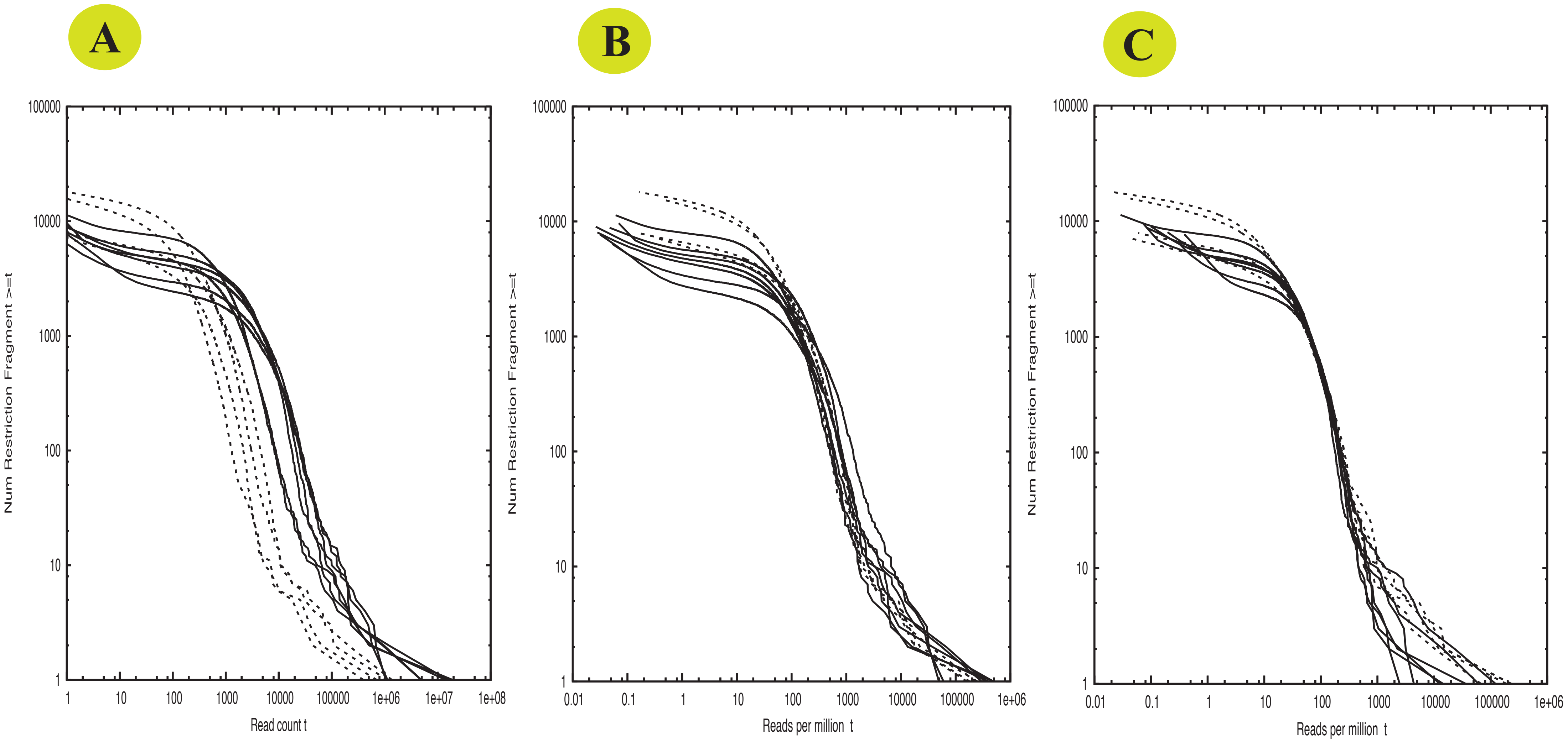

Supplementary Figure S1. Reverse cumulative distributions (logarithmic scale) of the number of restriction fragments that have at least one read mapped to them. The four dashed lines correspond to the four datasets generated using the *Myb* promoter as a 3C-seq viewpoint in fetal liver erythrocytes (FL) and fetal brain cells (FB). HindIII was used as a primary restriction enzyme. The seven solid curves correspond to seven datasets generated with various viewpoints in mouse pre-B cells, using BglII as a primary restriction enzyme. A) Reverse cumulative distributions before normalization. B) Reverse cumulative distributions after normalization with the simple RPM calculation method. C) Reverse cumulative distributions after normalization with the reverse-cumulative fitted values from the power-law distributions.

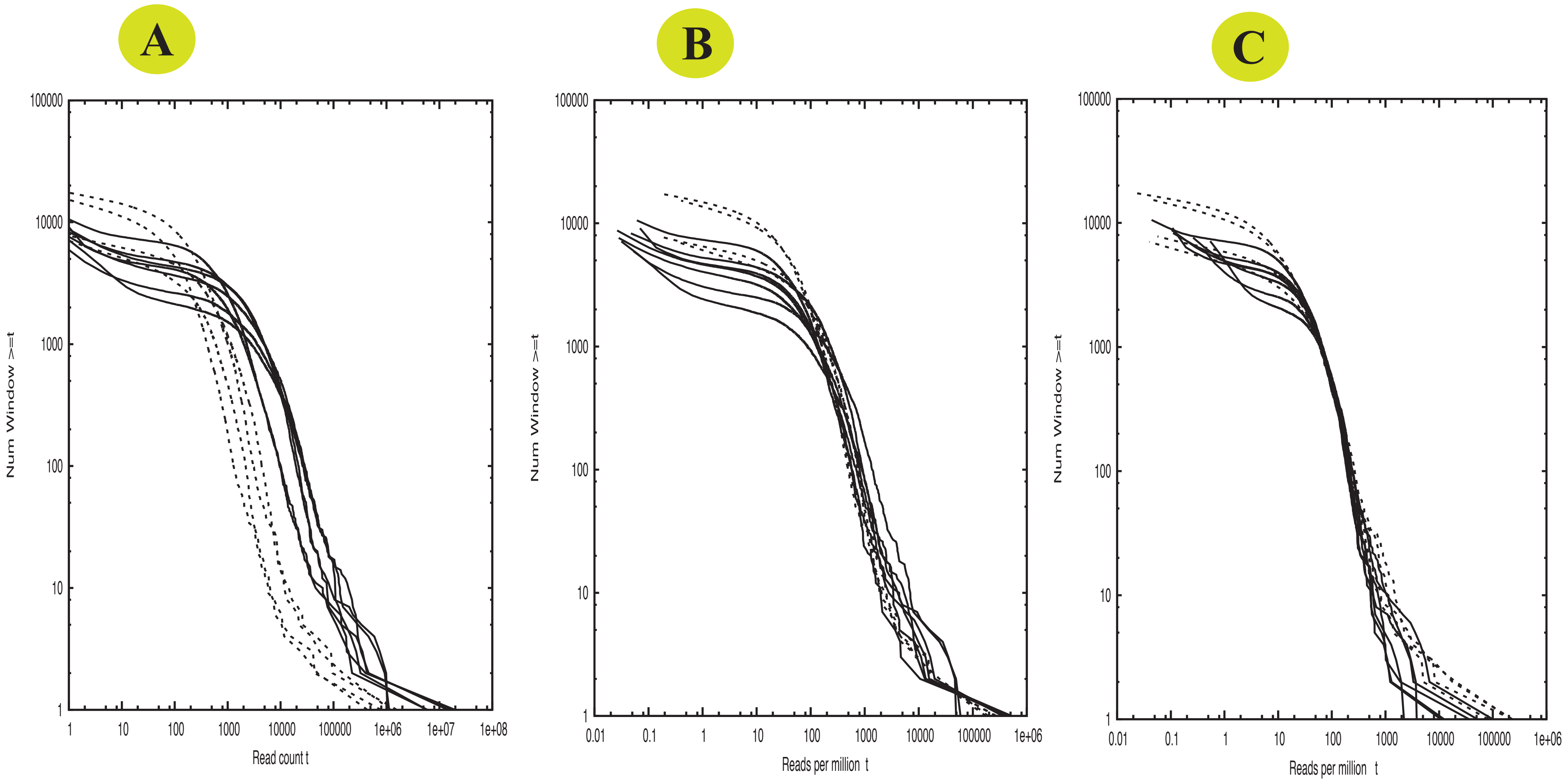

Supplementary Figure S2. Reverse cumulative distributions (logarithmic scale) of the number of 5 kb binning windows that have at least one read mapped to them. The four dashed curves correspond to the four datasets generated using the *Myb* promoter as a 3C-seq viewpoint in fetal liver erythrocytes (FL) and fetal brain cells (FB). The seven solid curves correspond to seven datasets generated with various viewpoints in mouse pre-B cells. A) Reverse cumulative distributions before normalization. B) Reverse cumulative distributions after normalization with the simple RPM calculation method. C) Reverse cumulative distributions after normalization with the reverse-cumulative fitted values from the power-law distributions.

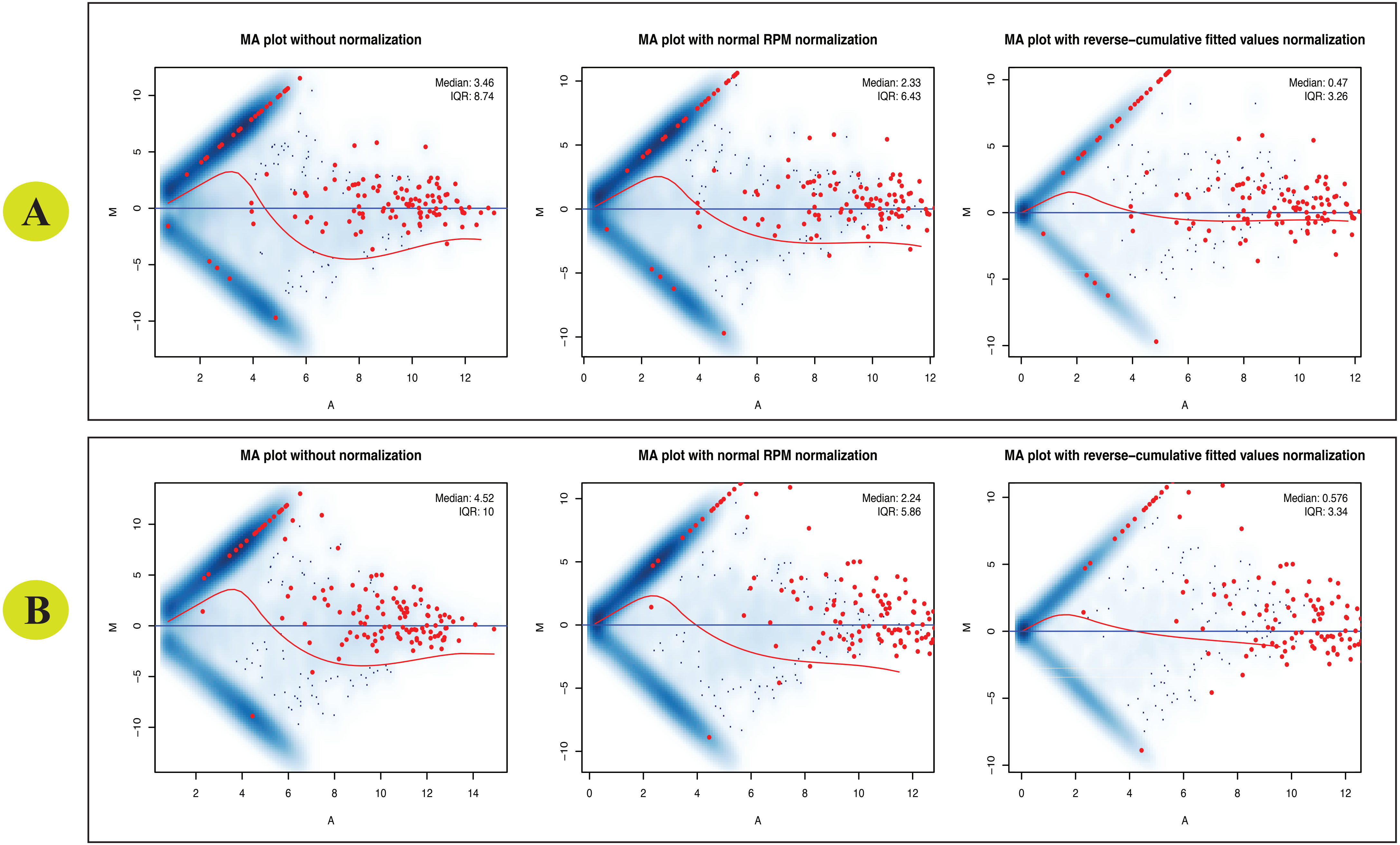

Supplementary Figure S3. The MA plots of the log2 intensity ratio (M) versus the average log2 intensity values (A) for two sets of independently prepared samples using no normalization, a simple RPM normalization, or the reverse-cumulative fitted values normalization. A) MA plots of data points generated using the Myb promoter as a 3C-seq viewpoint in fetal liver erythrocytes (FL) against fetal brain cells (FB) in biological replicate 1. B) MA plots of data points generated using the Myb promoter as a 3C-seq viewpoint in fetal liver erythrocytes (FL) against fetal brain cells (FB) in biological replicate 2. The red line represents the loess smoothing of M-A values and the red dots show the data points located within ±200 kb of the viewpoint.

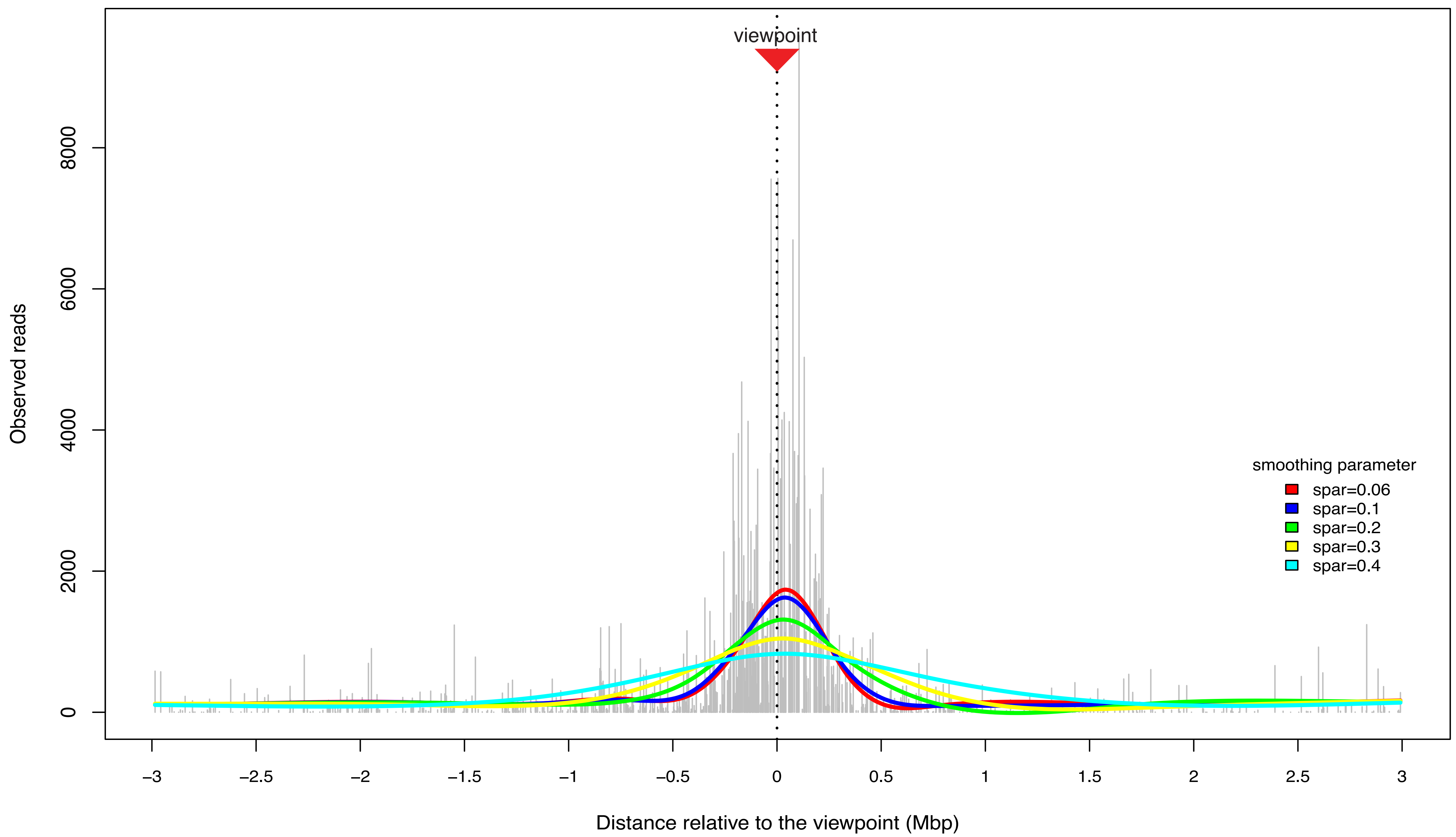

Supplementary Figure S4. Scaling of background interaction signals using a nonparametric regression cubic smoothing spline algorithm. Interaction signals with the *Myb* promoter viewpoint in fetal liver erythrocytes (FL) were ranked based on the relative genomic distance to the viewpoint. The plot shows the observed interaction signals and the scaled interaction signal using different smoothing parameters within a ±3 Mbp window around the viewpoint.

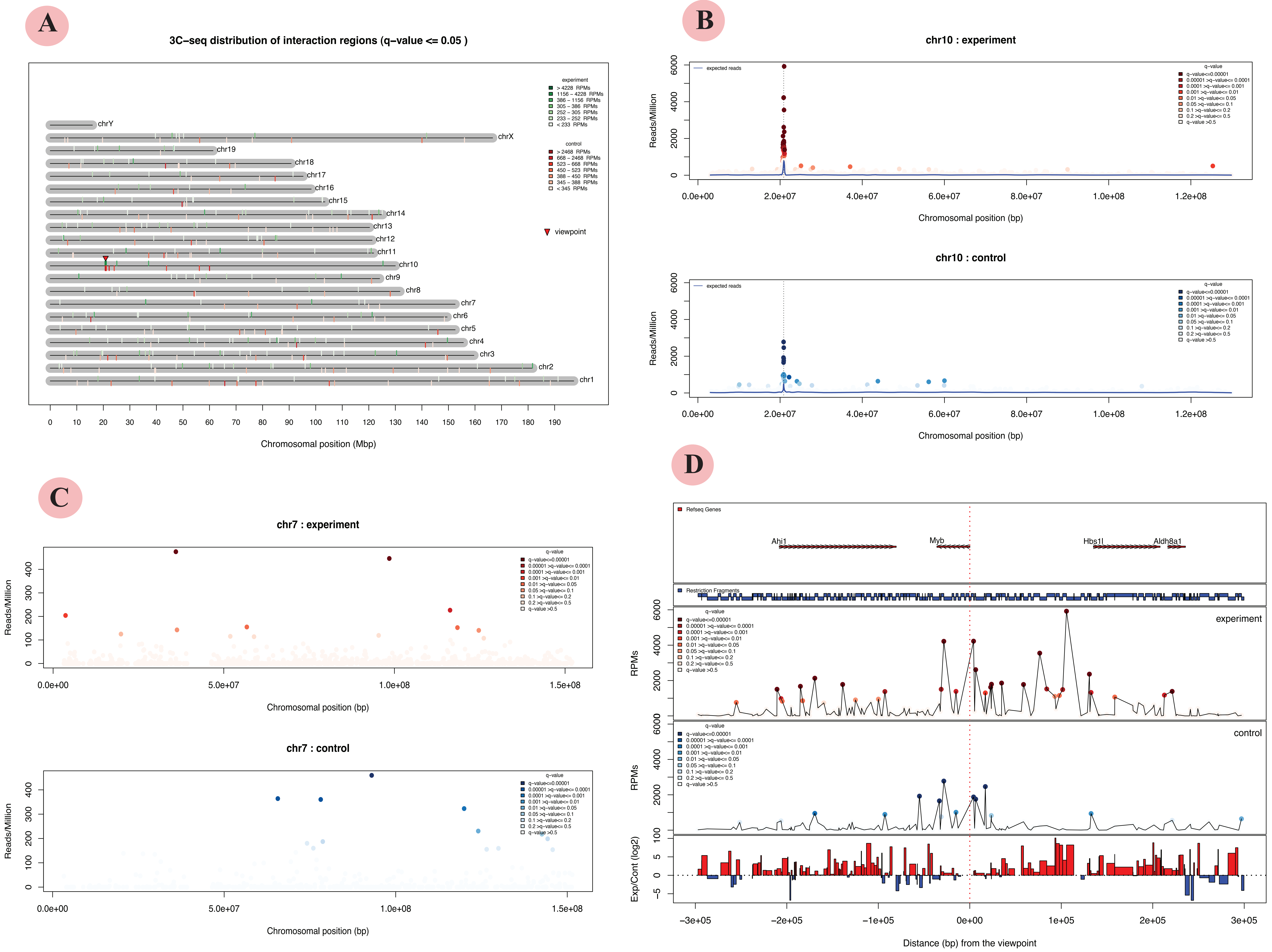

Supplementary Figure S5. Candidate interaction regions with the Myb promoter in both fetal liver erythrocytes (FL) and fetal brain cells (FB) explored in four different plots generated by *r3Cseq*. **A)** Plot generated by the plotOverviewInteractions function, which explores the distribution of candidate interaction regions on a genome-wide scale. **B)** Plot generated by the plotInteractionsPer-Chromosome function, showing the interaction regions along the selected *cis* chromosome. **C)** Plot generated by the plotInteractionsPerChromosome function, showing the interaction regions along the selected *trans* chromosome. D) Plot generated by the plotInteractionsNearViewpoint function, which is used to zoom in on the viewpoint region to visualize the distribution of interaction regions around it. It provides tracks with Refseq genes, restriction fragment locations, interaction signals of the experiment dataset, interaction signal of the control dataset and the ratio (log2) of normalized interaction signals between the experiment and control datasets.

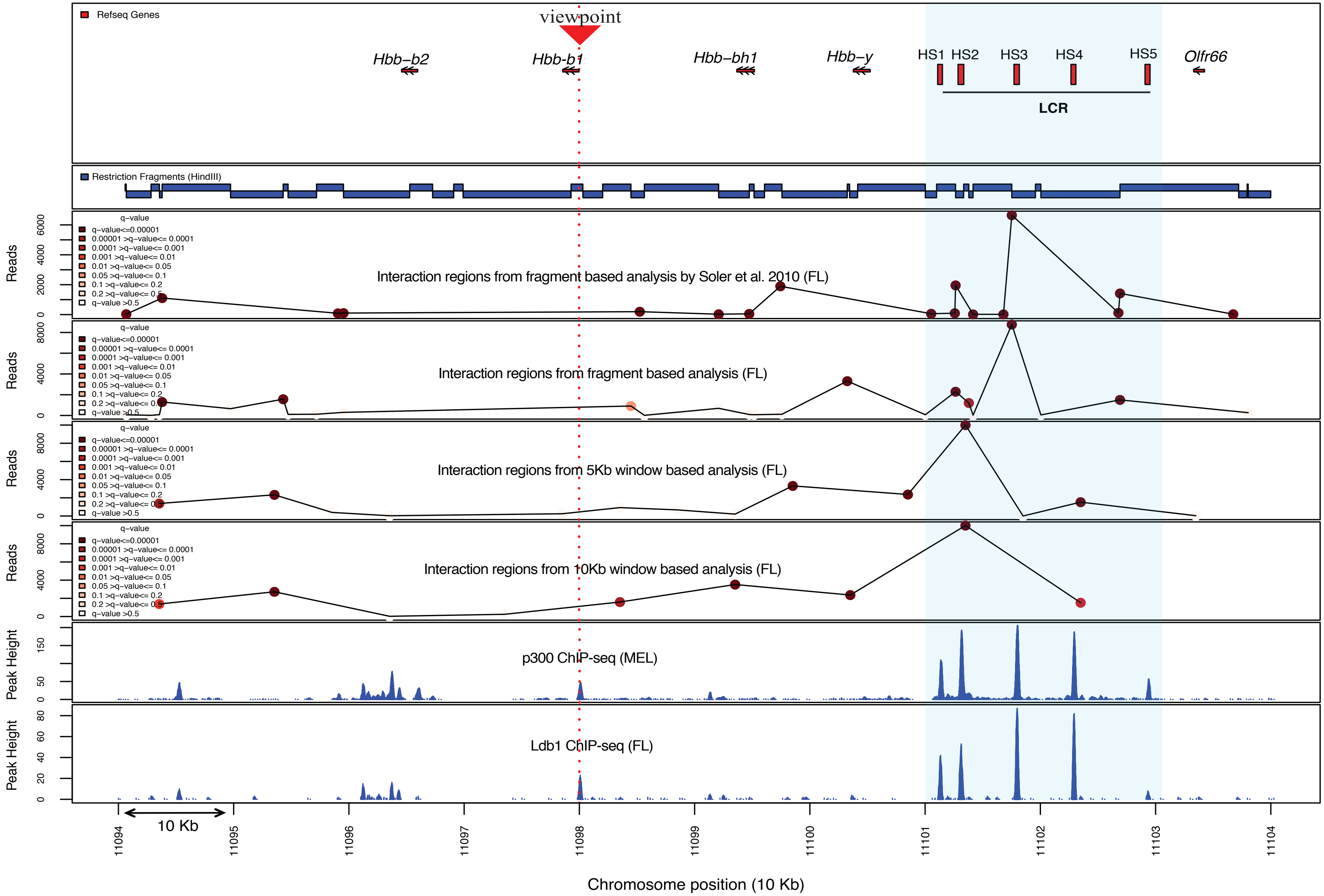

Supplementary Figure S6. Comparison of detected interactions using a restriction fragment based or window based analysis method for the β-maj promoter 3C-seq data from fetal liver erythrocytes (FL). Gene locations are shown at the top followed by a map of restriction fragments. The line plots show detected *cis*-interaction regions (40 kb up- and 60 kb downstream of the viewpoint) for either the fragment based approach used in our previous study, or using the *r3Cseq* fragment based, 5 kb window and 10 kb window based methods respectively. Color gradients represent the range of significant interaction signals (*q*-value). The light blue box highlights the LCR region with its hypersensitive sites (HS1-5) coinciding with several FL-specific significant interaction regions and binding sites of transcription factor complexes.

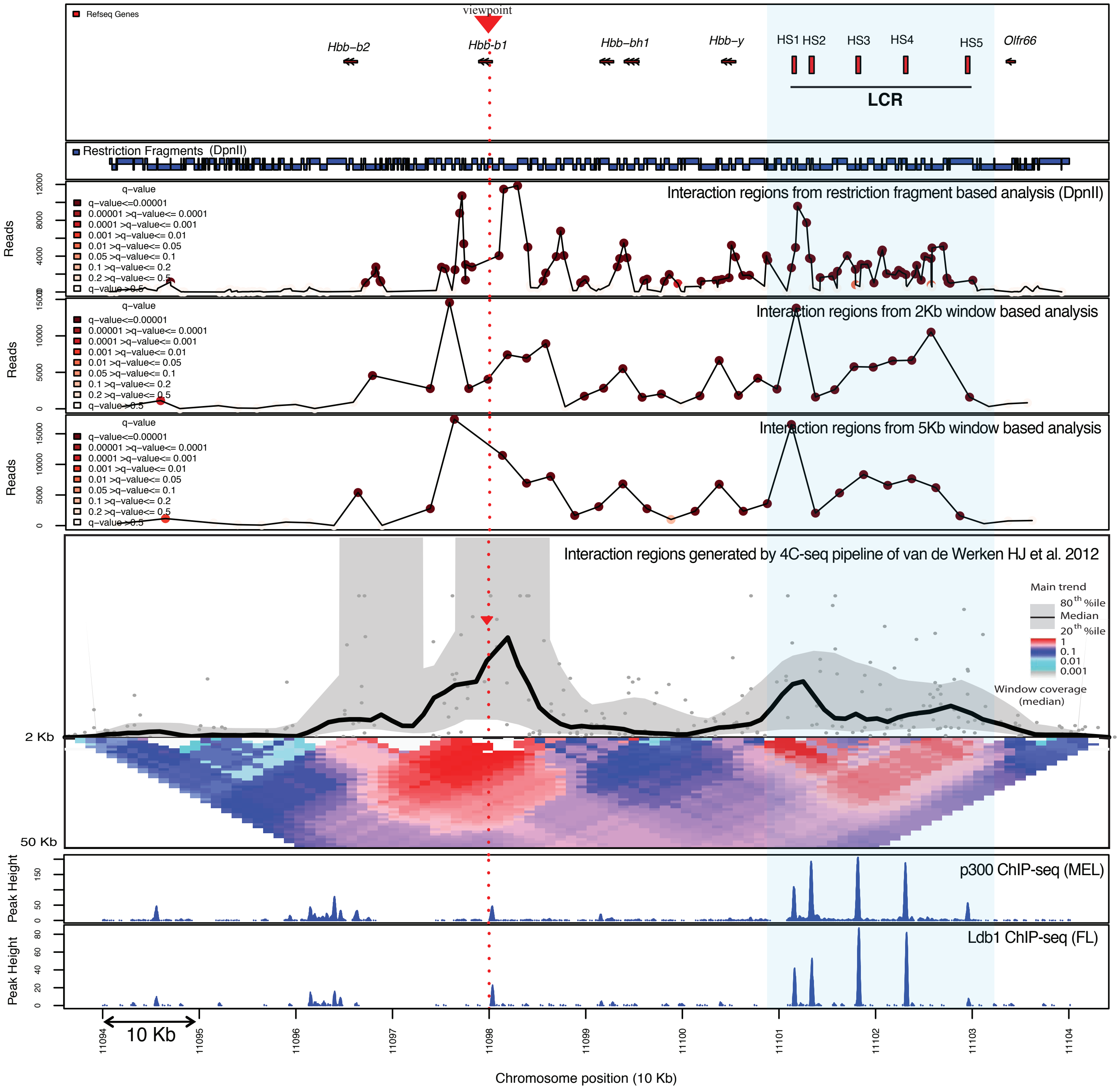

Supplementary Figure S7. *r3Cseq* analysis results using 4C-seq data generated by van de Werken HJ et al. using individual restriction fragments, 2 kb and 5 kb window-based analysis in the 100 kb β-globin domain. Gene locations are shown at the top followed by a map of restriction fragments. The line plots show detected cis-interaction regions (40 kb up- and 60 kb downstream of the viewpoint) for the fragment based, 5 kb window and 10 kb window based methods respectively. Color gradients represent the range of significant interaction signals (q-value). The domainogram was generated by the 4C-seq pipeline developed by van de Werken HJ et al. The light blue box highlights the LCR region with its hypersensitive sites (HS1-5) coinciding with several FL-specific significant interaction regions and binding sites of p300 and LDB1.

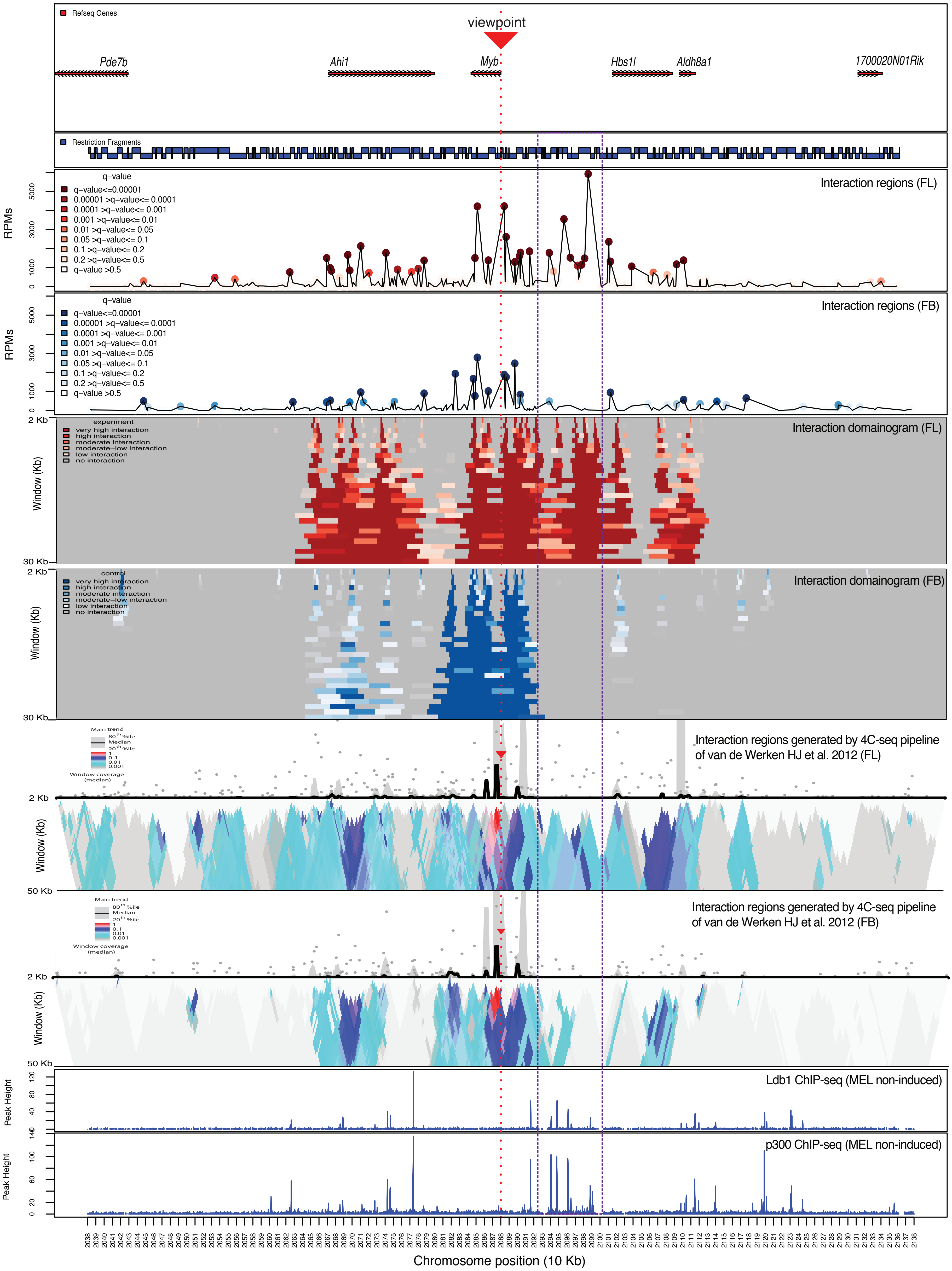

Supplementary Figure S8. Comparison of 3C-seq data analysis using both the *r3Cseq* package and the 4C-seq pipeline described in van de Weken HJ et al. using the *Myb* promoter data sets generated in both fetal liver erythrocytes (FL) and fetal brain (FB) cells. For the *r3Cseq* analysis, high signals on the viewpoint fragment or the immediately adjacent fragments were excluded. Line plots show detected *cis*-interaction regions 500 kb up- and 500 kb downstream of the *Myb* viewpoint. The domainograms show detected interactions using a window based analysis (running from 2 kb to 30 kb) for both datasets. Color gradients of domainograms represent the interaction signal strength detected for each run of the defined window (transformed *q*-value). For the 4C-seq pipeline analysis, domainograms were generated using default parameters provided by the software. Color gradients represent the normalized window coverage. The purple dashed box highlights the *Myb-Hbs1l* intergenic region, which shows strong and specific interaction signals in FL using both methods, coinciding with binding sites of LDB1 and p300.

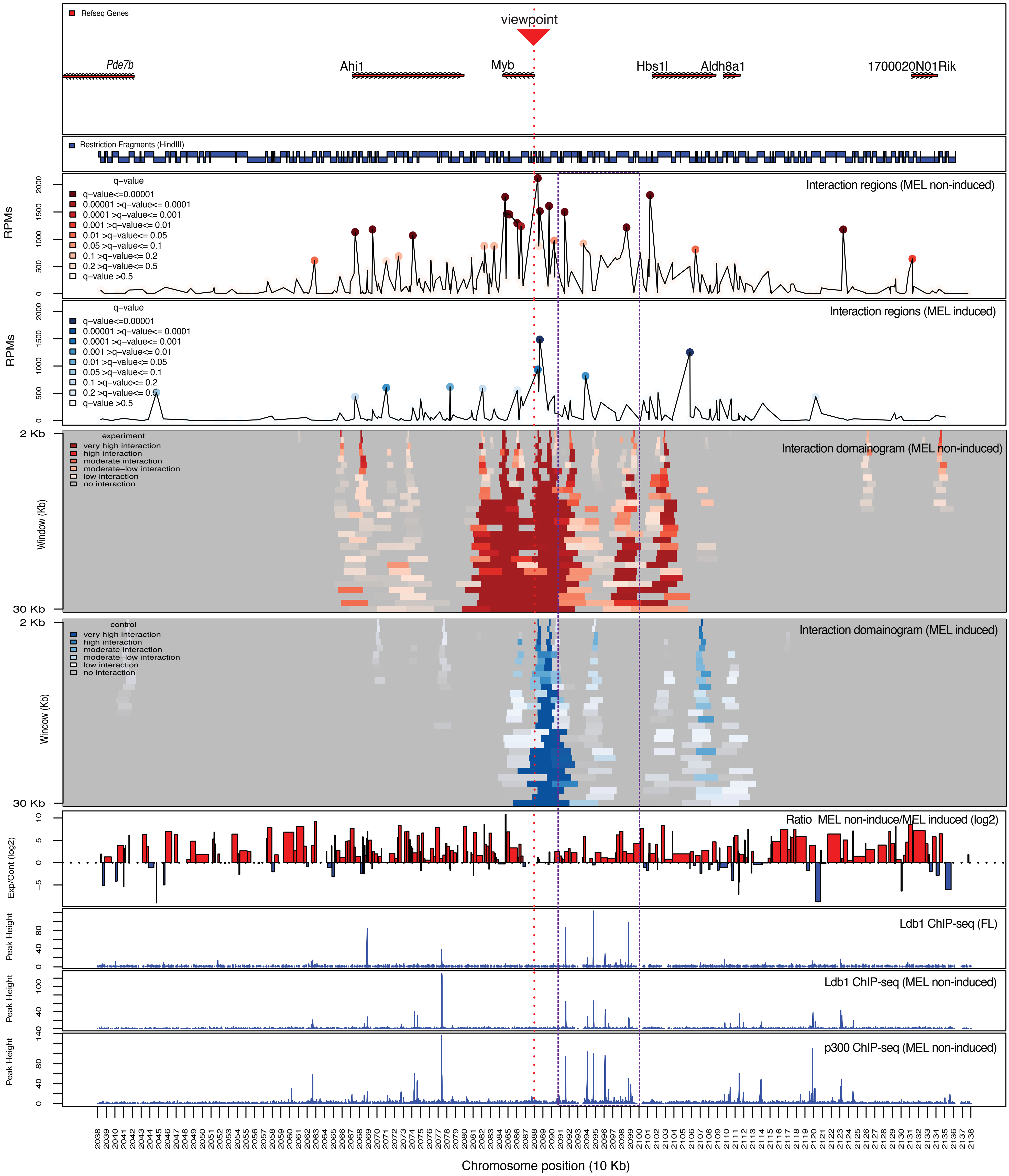

Supplementary Figure S9. Application of 3C-seq/*r3Cseq* to analyze chromatin interaction dynamics during erythroid differentiation at the *Myb* locus. Gene locations are shown at the top followed below by a map of restriction fragments. The line plots show detected *cis*-interaction regions 500 kb up- and 500 kb downstream of the *Myb* viewpoint in MEL cells before and after induction of differentiation. High interaction signal on the viewpoint fragment and those immediately adjacent have been excluded. Domainograms show detected interactions using a window based analysis (running from 2 kb to 30 kb) for both datasets. Color gradients of domainograms represent the interaction signal strength detected for each run of the defined window (transformed *q*-value). The purple dashed box highlights the *Myb-Hbs1l* intergenic region, which shows strong interaction signals coinciding with binding sites of LDB1 and p300.

A

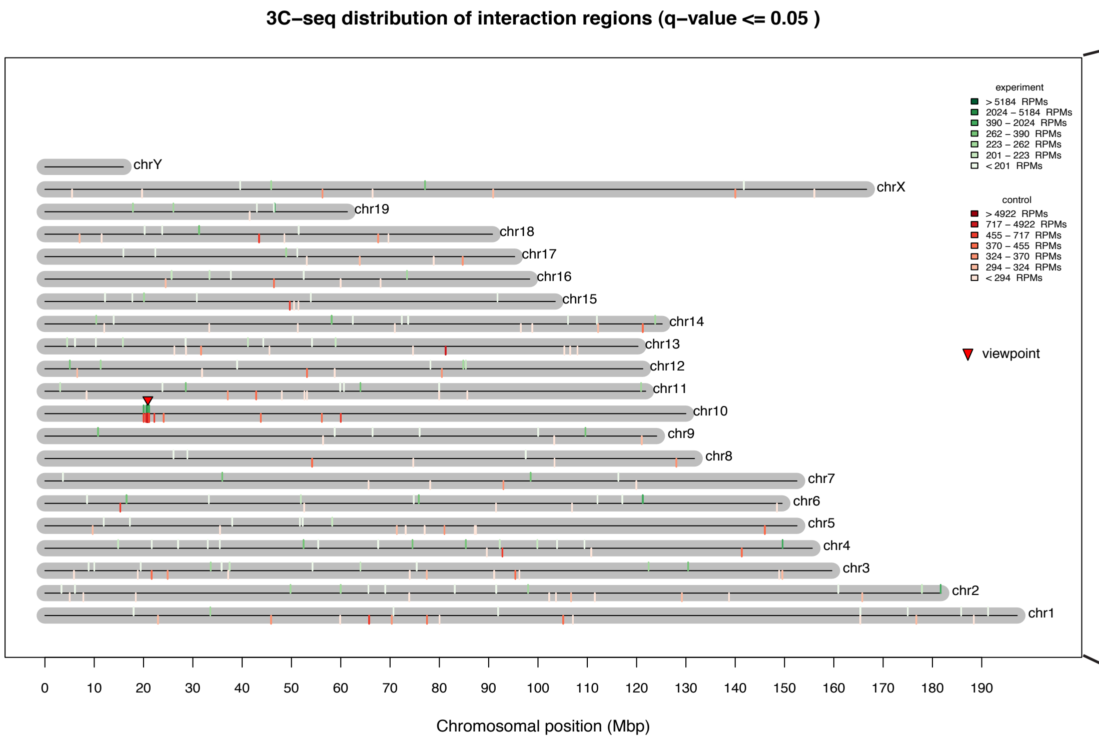

C

Chromosome	RefGene	Total nReads	Total RPMs
chr10	Ahi1	73119	19693.99
chr10	Myb	65116	19216.10
chr10	Hbs1l	63539	18128.22
chr10	Aldh8a1	28264	7230.41
chr4	Gm5506	12238	2276.72
chr4	BC002163	10311	1886.58
chr4	Gm5801	9915	1850.18
chr9	Cdkn2d	5901	1090.88
chr5	A430089I19Rik	5969	1057.97
chr5	AU018829	5969	1057.97
chr5	BC061212	5969	1057.97
chr5	E330014E10Rik	5969	1057.97
chr5	Gm16367	5969	1057.97
chr5	Gm3286	5969	1057.97
chr5	Gm6367	5969	1057.97
chr19	Snora19	5280	1002.80
chr4	4933409K07Rik	5196	929.03
chr4	Ccl21b	5196	929.03
chr4	Ccl21c	5196	929.03
chr4	Gm10591	5196	929.03
chr4	Gm13298	5196	929.03
chr4	Gm13304	5196	929.03
chr4	Gm13308	5196	929.03
chrX	Gm15085	4676	858.59
chrX	Gm15093	4676	858.59
chrX	Ott	4676	858.59
chr7	Mir684-1	4159	792.34
chr10	Bclaf1	3400	772.20
chr10	Fam54a	3400	772.20
chr17	Sult1c1	4213	756.44
chr5	Tff1	4061	722.91
chr2	Gm14496	2769	603.30
chr19	Sufu	2947	575.80
chr8	Tsnax	3039	540.42
chr7	Gm4535	2529	473.32
chr11	Gm12166	2430	450.01
chr6	Slc6a12	2123	438.33
chr6	Slc6a13	2123	438.33
chr19	Trim8	2049	420.03

B

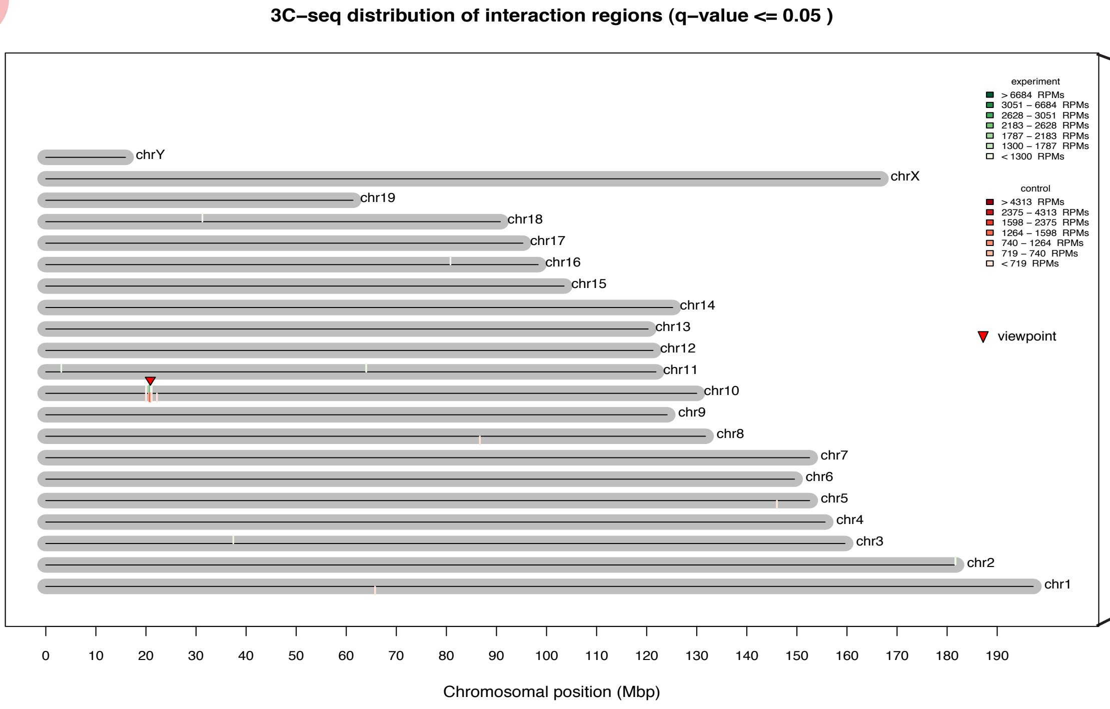

D

Chromosome	RefGene	Total nReads	Total RPMs
chr10	Myb	118188	19190.59
chr10	Ahi1	104470	16553.70
chr10	Hbs1l	90117	14978.97
chr10	Aldh8a1	35602	5551.43
chr10	Bclaf1	5502	717.83
chr10	Fam54a	5502	717.83
chr2	Gm14496	2477	379.04
chr11	Eif4enif1	1426	162.06
chr11	Sfi1	1426	162.06
chr3	Spata5	1382	156.91
chr18	Rit2	874	149.78

Supplementary Figure S10. Exemplary analysis comparing single sample analysis and analysis with replicates using the Myb promoter viewpoint datasets from fetal liver erythrocytes (FL) and fetal brain cells (FB). A) The genome-wide distribution of significant interaction regions for a single r3Cseq dataset. B) The genome-wide distribution of significant interaction regions after analyzing *r3Cseq* biological replicates. C) Top 40 genes found in close proximity to significant interaction signals in the FL erythrocytes of single sample analysis. D) Genes detected in close proximity to significant interaction signals present in FL erythrocytes of biological replicates.

Cis/Trans	Per region	Sample	Interactions present only in replicate1	Interactions present only in replicate2	Interactions present in common
cis-interactions	per restriction fragment	Myb viewpoint in fetal liver	1,499	1,888	591 (28% for replicate1 and 24% for replicate2)
	per 5 Kb window	Myb viewpoint in fetal liver	1,277	1,626	672 (34% for replicate1 and 28% for replicate2)
	per 10 Kb window	Myb viewpoint in fetal liver	1,065	1,294	727 (40% for replicate1 and 36% for replicate2)
	per restriction fragment	Myb viewpoint in fetal brain	831	1,041	208 (20% for replicate1 and 17% for replicate2)
	per 5 Kb window	Myb viewpoint in fetal brain	732	940	229 (24% for replicate1 and 20% for replicates2)
	per 10 Kb window	Myb viewpoint in fetal brain	675	850	226 (25% for replicate1 and 21% for replicate2)
trans-interactions	per restriction fragment	Myb viewpoint in fetal liver	7,791	9,227	178 (0.02% for both replicated1 and replicate2)
	per 5 Kb window	Myb viewpoint in fetal liver	7,923	9,280	349 (0.04% for both replicate 1 and replicate2)
	per 10 Kb window	Myb viewpoint in fetal liver	7,831	9,041	535 (0.06% for replicate1 and replicate2)
	per restriction fragment	Myb viewpoint in fetal brain	3,612	4,579	47 (0.01% for both replicates)
	per 5 Kb window	Myb viewpoint in fetal brain	3,622	4,570	76 (0.02% for both replicates)
	per 10 Kb window	Myb viewpoint in fetal brain	3621	4503	111 (0.3% for replicate1 and 0.02% replicate2)

Supplementary Table1. The calculated reproducibility of 3C-seq data from replicate experiments using fragment-based and window-based analysis methods (interaction signals >=1 RPM).

Cis/Trans	Per region	Sample	Interactions present only in replicate1	Interactions present only in replicate2	Interactions present in common
cis-interactions within ± 500 kb relative to the viewpoint	per restriction fragment	Myb viewpoint in fetal liver	16	4	21 (57% for replicate1 and 84% for replicate2)
	per 5 Kb window	Myb viewpoint in fetal liver	13	5	26 (67% for replicate1 and 84% for replicate2)
	per 10 Kb window	Myb viewpoint in fetal liver	12	3	28 (70% for replicate1 and 90% for replicate2)
	per restriction fragment	Myb viewpoint in fetal brain	6	3	6 (50% for replicate1 and 67% for replicate2)
	per 5 Kb window	Myb viewpoint in fetal brain	7	2	8 (53% for replicate1 and 80% for replicates2)
	per 10 Kb window	Myb viewpoint in fetal brain	6	2	10 (63% for replicate1 and 83% for replicate2)

Supplementary Table 2. The calculated reproducibility of 3C-seq data from replicate experiments using fragment-based and window-based analysis methods (interaction signals >=500 RPM).

Viewpoint	Experiment file name	Control file name	Experim ent file size (MB)	Control file size (MB)	Number of reads in experiment	Number of reads in the control	Processing time of *r3Cseq* (min)
Myb promoter	MYB_Prom_12.5_FL.bam	MYB_Prom_12.5_FB.bam	134	145	2,950,203	3,253,519	3.06
β-major promoter	Beta_Pro_FL.bam	Beta_Pro_FB.bam	248	296	6,281,237	7,241,107	5.77

Supplementary Table 3. Comparison of processing run times of the *r3Cseq* pipeline for different data sets. *r3Cseq* was performed using R version 2.15 running on a standard personal computer (MAC OS X with 2.53GHz Intel Core Duo and 4GB RAM).
